# The NeoPACE study: study protocol for the development of a core outcome set for neonatal palliative care

**DOI:** 10.1186/s12904-023-01326-x

**Published:** 2023-12-19

**Authors:** Katie Gallagher, Kathy Chant, Alex Mancini, Myra Bluebond-Langner, Neil Marlow

**Affiliations:** 1 UCL Elizabeth Garrett Anderson Institute for Women’s Health, University College London, 74 Huntley Street, WC1E 6AU London, UK; 2 Chelsea and Westminster Hospitals NHS Foundation Trust, London, UK; 3 UCL Institute for Child Health, University College London, London, UK

**Keywords:** Neonatal intensive care, Palliative care, Outcome studies

## Abstract

**Background:**

Neonatal death is the leading category of death in children under the age of 5 in the UK. Many babies die following decisions between parents and the neonatal team; when a baby is critically unwell, with the support of healthcare professionals, parents may make the decision to stop active treatment and focus on ensuring their baby has a ‘good’ death. There is very little evidence to support the clinical application of neonatal palliative care and/or end-of-life care, resulting in variation in clinical provision between neonatal units. Developing core outcomes for neonatal palliative care would enable the development of measures of good practice and enhance our care of families. The aim of this study is to develop a core outcome set with associated tools for measuring neonatal palliative care.

**Method:**

This study has four phases: (1) identification of potential outcomes through systematic review and qualitative interviews with key stakeholders, including parents and healthcare professionals (2) an online Delphi process with key stakeholders to determine core outcomes (3) identification of outcome measures to support clinical application of outcome use (4) dissemination of the core outcome set for use across neonatal units in the UK. Key stakeholders include parents, healthcare professionals, and researchers with a background in neonatal palliative care.

**Discussion:**

Developing a core outcome set will standardise minimum reported outcomes for future research and quality improvement projects designed to determine the effectiveness of interventions and clinical care during neonatal palliative and/or end-of-life care. The core outcome set will provide healthcare professionals working in neonatal palliative and/or end-of-life support with an increased and consistent evidence base to enhance practice in this area.

**Trial registration:**

The study has been registered with the COMET initiative (https://www.comet-initiative.org/Studies/Details/1470) and the systematic review is registered with the International Prospective Register of Systematic Reviews (PROSPERO) (CRD42023451068).

## Background

Neonatal units provide care to critically unwell preterm and term babies following birth, with approximately 1 in 7 newborns admitted for specialist care in the UK each year. Admission of a baby to a neonatal unit is extremely stressful for parents, where uncertainty around the prognosis of their baby can lead to complex ethical and emotional situations [1–3]. In 2020, 1,051 babies died during the neonatal period (the first 28 days of life), representing 1.53 deaths per 1,000 live births and accounting for the majority of deaths in children under 5 years in the UK [4, 5]. The main causes of death in neonates are complications of prematurity, congenital anomalies, infections or complications arising during labour [4, 6]. The recent MBRACE report highlighted the impact of ethnicity upon neonatal mortality rates, with the highest rates seen in babies of Pakistani, Black African and Bangladeshi ethnicity when compared to babies of white ethnicity. This was compounded by deprivation across all ethnic groups [6].

Palliative care is an active approach to improve the quality of life of patients and their families when facing health-related suffering associated with life-threatening, or chronic, illness [7]. A review of all data collected on all babies born and admitted to neonatal units in England and Wales between 2015 and 2020 identified that 2% of babies had palliative care needs, according to the categories identified in the British Association of Perinatal Medicine framework for palliative care [8]. Components of palliative care may include shared decision making, religious or spiritual support, alleviation of pain or discomfort, and advanced care planning, however will remain individual to each family [9]. Palliative care in neonatal settings can provide vital support for babies, parents and healthcare professionals by promoting goal planning and advocating for symptom management during all stages of the baby’s illness, including end-of-life [2, 10]. In neonatal settings when uncertainty may exist around the potential prognosis for an infant, many neonatal deaths are planned following continual review of the infants prognosis by parents and healthcare professionals and parallel curative and palliative care planning from birth or diagnosis [11, 12]. This allows for transparent and consistent information to be provided to best support and prepare parents for the potential death of their infant [9]. Some infants receiving palliative care in neonatal settings survive; the increase in prevalence of children with life-limiting or life threatening conditions is largest in children under the age of 1 year in the UK, highlighting the need for palliative care services for eligible infants from antenatal diagnosis or birth [13].

Despite national and international endorsement for palliative care there is little evidence to support the clinical application of neonatal palliative care, as clinical trials or large observation studies are not always appropriate in palliative care research for ethical, economic or practical reasons [10, 14–23]. This results in variation in inter-professional working, symptom management, decision-making practices, and parental psychological support despite parents being at high risk of severe anxiety and depression when facing the potential death of their baby [24–30].

In the absence of evidence from clinical trials the Delphi process has been adopted by clinicians and researchers in the field of palliative care to help develop guidelines, quality indicators, and measurable and comparable outcomes of care [14, 31–33]. Frequently employed in core outcome set (COS) development, the Delphi process gives key stakeholders the opportunity to prioritise the most important outcomes which should be measured and reported as a minimum in all future research in a specific area [31, 34]. The development of COS are supported by both the Core Outcome Measures in Effectiveness Trials (COMET) initiative (www.comet-initiative.org) and the Core Outcomes in Women’s and Newborn Health (CROWN) initiative, in an attempt to minimise outcome reporting bias across researchers, facilitate study outcome comparison, and ensure outcomes relevant to all stakeholders are considered in all future studies [35–37].

The aim of this study is to develop a COS and associated measurement tools for neonatal palliative care. Central to the COS is the views of key stakeholders including parents from different ethnic backgrounds, to ensure that final recommended outcomes are relevant to all families in neonatal settings, healthcare professionals, and researchers.

## Methods

**Fig. 1:**
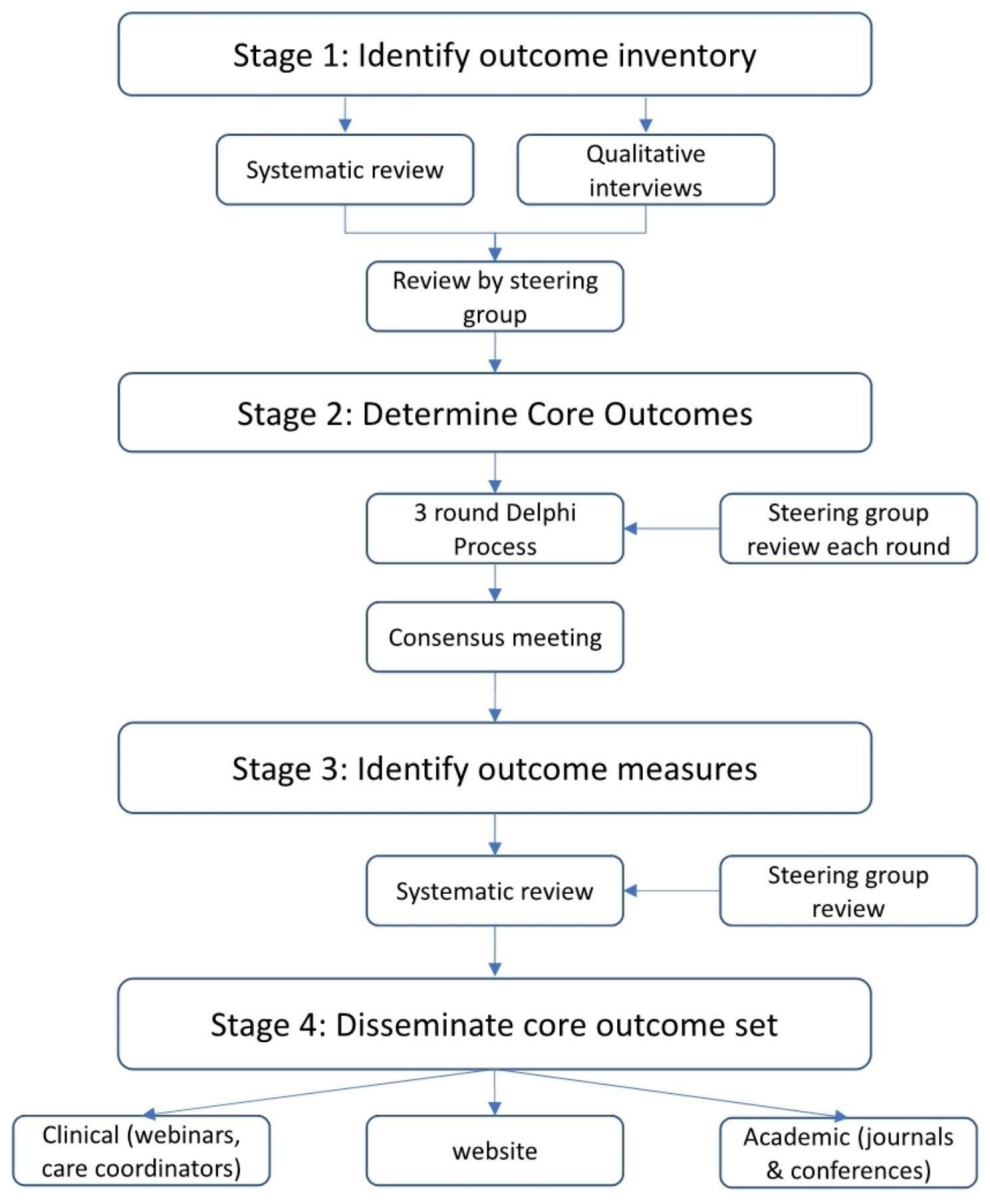
Stages of development of the core outcome set for neonatal palliative care

The methodology to support the development of the core outcome set for neonatal palliative care (the NeoPACE study) will be guided by the recommendations from the COMET handbook and the Core Outcome Set Standards for Protocol items (COS-STAP), Development (COS-STAD), and Reporting (COS-STAR), adapted to the study scope [34, 38–40]. The study will be completed in 4 stages: [1] identification of potential outcomes through systematic review and qualitative interviews with key stakeholders, [2] determining core outcomes through an online Delphi process with key stakeholders, [3] identification of appropriate outcome measures to support outcome use in clinical practice, [4] disseminating the core outcome set for use throughout the United Kingdom (Fig. 1).

### Steering group

A study steering group will be established to guide the development of the core outcome set. The steering group will consist of stakeholders from the following areas: neonatologists, obstetricians, neonatal nurses, palliative care specialists, and parent representatives. A Parent Advisory Group (PAG) will be formed by parents from neonatal and maternity parent representative and advocacy groups. This includes the Stillbirth and Neonatal Death Society, Together for Short Lives, Global Black Maternal Health, and The Motherhood Group. The steering group and PAG will be involved in all aspects of study design including defining the scope of the COS, reviewing, and refining the initial inventory of outcomes, preparing participant facing materials for recruitment (including animation design), reviewing the outcomes following the Delphi process, and developing the dissemination strategy.

### Study registration

The study has been registered with the COMET initiative (https://www.comet-initiative.org/Studies/Details/1470) and the systematic review is registered with the International Prospective Register of Systematic Reviews (PROSPERO) (CRD42023451068). The study has received ethical approval by the University College London (UCL) Research Ethics Committee (REC ID: 16,059/012).

### Scope of the core outcome set

The COS will be relevant to all babies eligible for palliative care and who are admitted to a neonatal care unit. This includes infants where an antenatal diagnosis has taken place, infants receiving end-of-life care on the neonatal unit or following transfer to home or hospice, and infants transitioning into paediatric palliative care services. The COS will cover all interventions provided as part of palliative and/or end-of-life care, including but not limited to physical comfort, parental support, decision making, and organ donation. The COS will be designed to enhance clinical practice, provide an ability to benchmark practice through audit, and identify the minimum reported outcomes in future neonatal palliative care research. It will be developed initially for the UK only, as service provision and access vary globally. On-going research will explore adaptation to other healthcare settings.

### Stage 1: identification of potential outcomes

A systematic review will be undertaken with the aim of developing an inventory of all outcomes reported in neonatal palliative care research. The search strategy will be developed with the support of a subject specific librarian using defined MeSH descriptor headings and undertaken in all relevant databases, with no date or language limitations. Academic articles not written in English will be translated either through the hosting search database such as EBSCOHost or following upload to Google translate. We anticipate including all trials and observational studies reporting an outcome following an intervention in neonatal palliative care.

All studies meeting the inclusion criteria will be reviewed by 2 reviewers independently (KG and KC) and any discrepancies discussed with a third (MB-L). Data will be extracted into a pre-designed outcome proforma. The methodological quality of both the studies and the outcome description and reporting in each study will be assessed using either the six-point reporting criteria developed for the Management of Otitis Media with Effusion in Cleft Palate (MOMENT) study or the CASP tool for qualitative research [41, 42]. A list of all outcomes from the systematic reviews will be compiled into an outcome inventory ready to integrate with the outcomes identified from the qualitative interview study.

### Assessment of outcomes important to key stakeholders

Parents^1^ and healthcare professionals affected by neonatal palliative care are essential in the development of the core outcome set and the identification of initial outcomes, as they may identify outcomes that are not recognised in existing literature [43, 44]. Qualitative methods are recommended to capture these outcomes, enhance research quality and the prospect of subsequent implementation success [34, 38, 45–47]. Semi-structured interviews following informed consent with parents and healthcare professionals will therefore be undertaken to explore their perceptions of outcomes important in neonatal palliative care and end-of-life care. Parents will be eligible for the study if, their:

1. Baby was diagnosed with a congenital anomaly during pregnancy and died either in the delivery suite or following admission to a neonatal unit.
2. Baby was admitted for neonatal care and died either in the neonatal unit, or following transfer to home, or a hospice.
3. Baby received care on the neonatal unit for a life limiting condition and is currently receiving palliative care at home, in a hospice, or a paediatric setting.

Parents will not be eligible if:

1. They have experienced the death of a baby within the previous 6 months, or more than 5 years previously.
2. They still have a baby admitted to the neonatal unit, or who has been discharged within 3 months.
3. Their infant died of sudden infant death syndrome (SIDS).
4. They live outside of the United Kingdom.
5. They are unable to consent or are under the age of 18.
6. They are unable to communicate in English (for the interview part of the study only).

There will be particular emphasis on ensuring study advertisement and recruitment of parents identifying as Black, Asian and Ethnic Minority as parents from this group are disproportionally affected by neonatal death however are often underrepresented in research; this study seeks to ensure the views of all potential parents are incorporated into the resulting core outcome set. We aim to recruit 24 parents through relevant neonatal, and maternal bereavement support charities. The study will be advertised through the social media channels of charities including the Stillbirth and Neonatal Death Charity (Sands), Together for Short Lives, The Motherhood Group, and Global Black Maternal Health. All participant facing materials will be developed in collaboration with the study parent advisory group, with support provided to all parents pre and post interview by specialist neonatal charities. Researchers on the project have experience with working with, and interviewing, bereaved neonatal families and are aware of when and how to signpost parents to receive further support.

Healthcare professionals will be eligible to participate in the interviews if they have relevant clinical experience in neonatal palliative and or end-of-life care and are currently working in the UK. Study information will be shared through the social media channels of the British Association of Perinatal Medicine (BAPM) and the Neonatal Nurses Association (NNA). Participants (n = 21) will be stratified by professional group (e.g. neonatal doctors, neonatal nurses, obstetricians, midwives, perinatal or neonatal palliative care specialist, bereavement counsellors) with three professionals recruited from each group (n = 18) through professional neonatal organisations. We will also recruit researchers (n = 3) active in the field of neonatal palliative and/or end-of-life care (identified from the systematic review).

Interviews will be conducted either in person or virtually, at the participants preference, and audio recorded either using a digital recorder or MSTeams. Audio recordings will be transcribed manually and MSTeams transcripts downloaded and checked for accuracy, prior to all transcripts being uploaded into NVivo for data management and analysis. Data will be analysed using a modified means of thematic analysis, identifying potential outcomes of neonatal palliative care whilst simultaneously grouping codes together into themes reflecting specific areas of discussion.[5, 94] Codes will be reviewed between researchers to increase validity of the analysis and reduce potential for lone researcher bias.[5] A merged inventory of outcomes will be developed, incorporating outcomes identified through the interviews and the systematic review. Where possible, outcomes will be placed into themes which reflect recommendations from the European Association of Palliative Care (physical, psychological, social and cultural, spiritual) [48, 49]. Amendments will be made to these themes if the outcomes identified for this subject area do not fit these particular categories.

### Stage 2: determining core outcomes

The core outcomes will be identified using an online Delphi process, which involves repeated reflection and re-scoring of potential outcomes to develop convergence of opinion, from which core outcomes can be determined [34, 50]. Key stakeholders will represent a wide group of parents and healthcare professionals affected by, or working clinically in the area of neonatal palliative and/or end-of-life care. To avoid under-representation of parents and the risk that the final COS is weighted towards a particular stakeholder group, two Delphi process will be undertaken; one for parents and parent representatives, and one for healthcare professionals and researchers [34].

Delphi study participants are not always sure of the purpose of a COS or the Delphi Process, and so a short, sensitive, animation (with subtitles to support participants whose first language is not English) explaining the Delphi process, the wider study aim and signposting to relevant support charities will be developed to facilitate participation [51]. This animation, along with wider study details (including the participant information sheet) will be shared widely through newsletter, email, and social media channels of neonatal and maternity support charities and neonatal professional organisations. The research team will also be available to support participants to complete the Delphi process through one-to-one support, in personal or virtually, if required.

### Round one

The survey will be hosted by DelphiManager, a web-based system designed specifically to facilitate Delphi surveys, facilitating participation and providing feedback between Delphi survey rounds [52]. DelphiManager can facilitate survey translation to enable participation from non-English speaking participants. Interested participants will be invited to register with DelphiManager, details of which will be included in the study information email. Participants will be asked to provide basic demographic information to determine their affiliation with a particular stakeholder group and will be allocated a unique identifier to ensure anonymity. Following registration, participants will receive an email from the research team via the DelphiManager software inviting them to complete the first round of the Delphi Survey.

Participants will be invited to score the importance of each outcome using a Likert scale between 1 and 9 [53]. Scores between 1 and 3 will indicate limited importance of the outcome, 4–6 important but not critical and between 7 and 9 as critically important. There will also be an ‘unable to rate’ box. All outcomes presented in the Delphi process will include a plain language definition measured by the Flesch-Kincaid Index, and a more detailed definition if appropriate. At the end of the survey participants will be able to add any additional outcomes they feel have been missed. The survey will remain open for 6 weeks with a weekly email remainder sent through the DelphiManager software to encourage completion.

At the end of round 1, a meeting will be held with the study steering group to determine whether any outcomes can be ‘dropped’ between round 1 and round 2 [34]. It will take into account whether the majority of participants across all stakeholder groups have already reached consensus on any outcomes using pre-defined criteria of when (across all stakeholder groups) greater than 70% of participants score an outcome between either 1–3 or 7–9, and less than 15% score the item between 7 and 9 and 1–3 respectively. Any additional outcomes which are added as part of a free text response during the first two rounds will be added to the subsequent round of both panel groups, to ensure that all potential outcomes are considered by all participants. Results will be combined in the consensus meeting to determine the final core outcome set.

### Round two

Only participants who have taken part in round one will be invited to participate in round two. Group and individual responses will be summarised and fed back anonymously to participants via DelphiManager, allowing them to reflect on the views of others before re-rating (if they wish) each outcome [34]. Following round two, the steering group will review the scores as before, deciding which outcomes can be dropped or carried into round three. If consensus has been reached, a third round will not be required. At the end of round two, participants will be asked to indicate their interest in attending a consensus meeting to finalise the COS, either following round two or round three.

### Round three

Participants who have taken part in round two will be invited to take part in the third and final round, to identify the core outcomes, if consensus has not been reached. Participants will be presented with the percentage distribution of scores across all outcomes as in round 2 and given the option to re-score the outcome if they wish. The predefined consensus criteria will be applied to this round to determine any outcomes meeting the criteria for in or out and reduce the final list for prioritisation. If neither criterion are met, an outcome will be classed as ‘no consensus’ and will be discussed further in the consensus meeting, of which confirmation of interest in attending will be confirmed. Attrition between rounds will also be assessed to determine the degree of loss in different participant groups.

The number of participants to include in a Delphi study is not based upon statistical power but rather ensuring the views of all stakeholders are captured and considered [34, 54–58]. Considering attrition, we aim to recruit 150 parents and/or parent representatives in panel 1, and 30 participants per stakeholder group in panel 2 (groups: neonatal, antenatal, palliative care specialists, counsellors, researchers: 150 participants). If recruitment fails to meet these numbers, prior to the Delphi study the steering group will review the invitation process, readvertising where possible to enhance participation. There will be no maximum number; if stakeholder numbers are higher from one particular group, a weighted percentage will be considered to ensure results are representative.

### Stakeholder meeting

Following the Delphi process a consensus meeting will be held with the steering committee and interested stakeholders to finalise the core outcome set, definitions and target population for each outcome [50, 59]. Breakout groups will be facilitated by a steering group member, with results from the Delphi forming the basis of the discussion. Groups will be asked to present their final list of outcomes in order of importance. The top 10 outcomes will be considered, to ensure that the final list can be implemented in clinical practice. If consensus on the top 10 cannot be achieved between groups, a final anonymous vote will determine the final core outcome set. Any items which do not reach consensus will be removed from the final core outcome set.

### Stage 3: determining how core outcomes should be measured

To enable future research comparison, different studies must measure the agreed outcomes in the same way. Outcome measures will therefore be identified using structured guidelines to determine how and when to measure the core outcomes [60–62]. Systematic review of each outcome will be undertaken to identify Outcome Measurement Instruments (OMI) previously used in clinical practice. An evaluation of the methodological qualities of the studies and the measurement properties will be undertaken using the COnsensus-based Standards for the selection of health Measurement INstruments (COSMIN) guidelines [62–64]. If no high-quality outcome measurements exist, further research will be explored to determine how to improve the evidence base. Evidence will be evaluated to provide an overall assessment of each measurement quality [53]. All measurements and their grading will be presented at a measurement meeting with the study steering group to finalise tools for recommendation.

### Stage 4: disseminating the core outcome set for use throughout the UK

Following on from the animation developed to facilitate participation, a second short animation will be developed to highlight the study results, using lay language and subtitles to ensure accessibility to non-English speaking populations. Dissemination of the animation, and the results, will be supported through the development of the first specialist, dedicated website for neonatal palliative and end-of-life care (www.neonatalpalliativecare.org.uk). The website link will be shared with all study participants, through neonatal and maternity organisations, professional neonatal, midwifery and obstetric organisations, using newsletters, emails, and social media strategy. The website will host resources around the outcomes and associated measurements to encourage clinicians and researchers to incorporate the core outcome set into future clinical trials and quality improvement. We will share our findings with the neonatal care coordinators in each neonatal network, to facilitate knowledge transfer to all neonatal units. We will work with relevant professional organisations to host webinars to also share our findings, to reach as wide an audience as possible. We will also share our results in relevant academic journals and conferences. Future research will pilot the use of the COS in clinical practice to determine the validity of the tool and how this can enhance neonatal palliative care provision.

## Discussion

Developing a core outcome set for neonatal palliative and end-of-life care will standardise minimum reported outcomes for future research and quality improvement projects designed to determine the efficacy of care provided during this extremely challenging time. Ensuring that the research represents the views of parents and healthcare professionals, in particular parents at higher risk of neonatal death in Black and Asian communities, will make certain that these outcomes reflect the experiences and opinions of a diverse range of families. The core outcomes allow for additional outcomes to be identified in future studies, whilst providing neonatal healthcare professionals with an increasing evidence base from which to improve practice [65]. Ultimately, our goal is to enhance equity and provision of neonatal palliative and end-of-life care to all families across the UK. Future work can then explore adaptation to other countries where the need for neonatal palliative care exists.

## Data Availability

Only the direct study team (KG and KC) will have access to the final data set. Access to the raw data will be controlled by the data information asset owner (KG).

## Footnotes

1 defined as mother and significant other, or those legally assigned care of the infant at the time decision making around palliative or end-of-life care undertaken.

## Abbreviations

BAPM: British Association of Perinatal Medicine
COMET: Core Outcome Measures in Effectiveness Trials
COS: Core outcome set
COSMIN: Consensus-based Standards for the selection of health measurement instruments
COS: STAD-Core Outcome Set Standards for Development
COS: STAP-Core Outcome Set Standards for Protocol items
COS: STAR-Core Outcome Set Standards for Reporting
CROWN: Core Outcomes in Women’s and Newborn Health
MOMENT: Management of Otitis Media with Effusion in Cleft Palate criteria
NNA: Neonatal Nurses Association
OMI: Outcome Measurement Instruments
UK: United Kingdom
